# Granulomatosis with polyangiitis: A case report and brief review of literature

**DOI:** 10.1016/j.radcr.2021.08.028

**Published:** 2021-09-05

**Authors:** Dhairya A Lakhani, Aneri B Balar, Ayodele Adelanwa, Alexander Gross, Rehab Mohamed, Kelly T Smith, Cathy Kim

**Affiliations:** a Department of Radiology, Diagnostic Radiology Residency Program, Morgantown, WV 26506, USA; b Department of Pathology, Anatomy and Laboratory Medicine; West Virginia University, Morgantown, WV 26506, USA

**Keywords:** Granulomatosis with polyangiitis, Vasculitis, Wegener's granulomatosis

## Abstract

Granulomatosis with polyangiitis formerly known as Wegener's granulomatosis was first described by German pathologist Friedrich Wegener in 1936. It is a multi-system necrotizing noncaseating granulomatous vasculitis which affects small to medium-sized vessels. It can involve any organ system, most commonly the lungs and kidneys. American College of Rheumatology requires 2 of 4 criteria for diagnosis: Positive biopsy for granulomatous vasculitis, urinary sediment with red blood cells, abnormal chest radiograph and oral/nasal inflammation. Here we present a case of Granulomatosis with polyangiitis with brief review of literature.

## Background

Granulomatosis with polyangiitis is a multi-system necrotizing vasculitis which typically involves respiratory system and kidneys [1], [2], [3], [4]. Radiographic presentation of pulmonary manifestations is categorized into four patterns [5], [6], [7], [8]. Most common radiologic presentation includes multiple nodules of various sizes in random distribution throughout the lungs with or without central cavitation [6], [7], [8]. Second most common pattern includes multifocal regions of consolidation or ground glass opacities reflecting pulmonary hemorrhage [6], [7], [8]. Reticulonodular pattern and peripheral wedge-like consolidation have also been described in the literature. Pleural effusion is reported in 10%-25% of cases. Tracheal and upper respiratory tract thickening may also be seen [6], [7], [8].

Renal manifestations of granulomatosis with polyangiitis tend to be occult on imaging [9,10]. Approximately 50% of these patients have kidney disease at clinical presentation, which are characterized by reduced renal function, proteinuria and hematuria [9,10]. Upper respiratory tract manifestations are common and presentation most commonly includes nasal obstruction, rhinitis and epistaxis. It manifests as sinusitis, mastoiditis, otitis, sinonasal mucosal ulcers, nasal septal perforation, nasal saddle deformity or subglottic stenosis. Enhancing soft tissue nodules in the sinonasal mucosa have also been described in the literature [4,10,11]. Central nervous system (CNS) manifestations are reported and approximately 5% of the patients. CNS manifestations include hypertrophic pachymeningitis, small vessel CNS vasculitis resulting in infarcts and/or arterial occlusion, and intracranial hemorrhage [4]. Ophthalmic manifestations have been reported in 40%-50% of patients [4]. It results in an orbital granulomatous noncaseating inflammatory mass often associated with proptosis with or without optic nerve compression. Small vessel vasculitis component may result in conjunctivitis, scleritis, episcleritis, uveitis, optic neuritis, optic nerve vasculitis or retinitis [3,4].

Here, we present a case of Granulomatosis with polyangiitis presenting with pulmonary and upper respiratory tract manifestations.

## Case presentation

The patient is a 20-year-old Caucasian male with no past medical history presents to the emergency department with low grade fever, left ear pain and headache for 4 weeks. Review of systems was positive for dry cough and sinus disease. On presentation, patient was febrile to 100.7 Fahrenheit (38.2 degrees Celsius), heart rate of 110, blood pressure of 114/63 and oxygen saturation 94%. Physical examination revealed mild tenderness at maxillary sinuses.

**Fig. 1:**
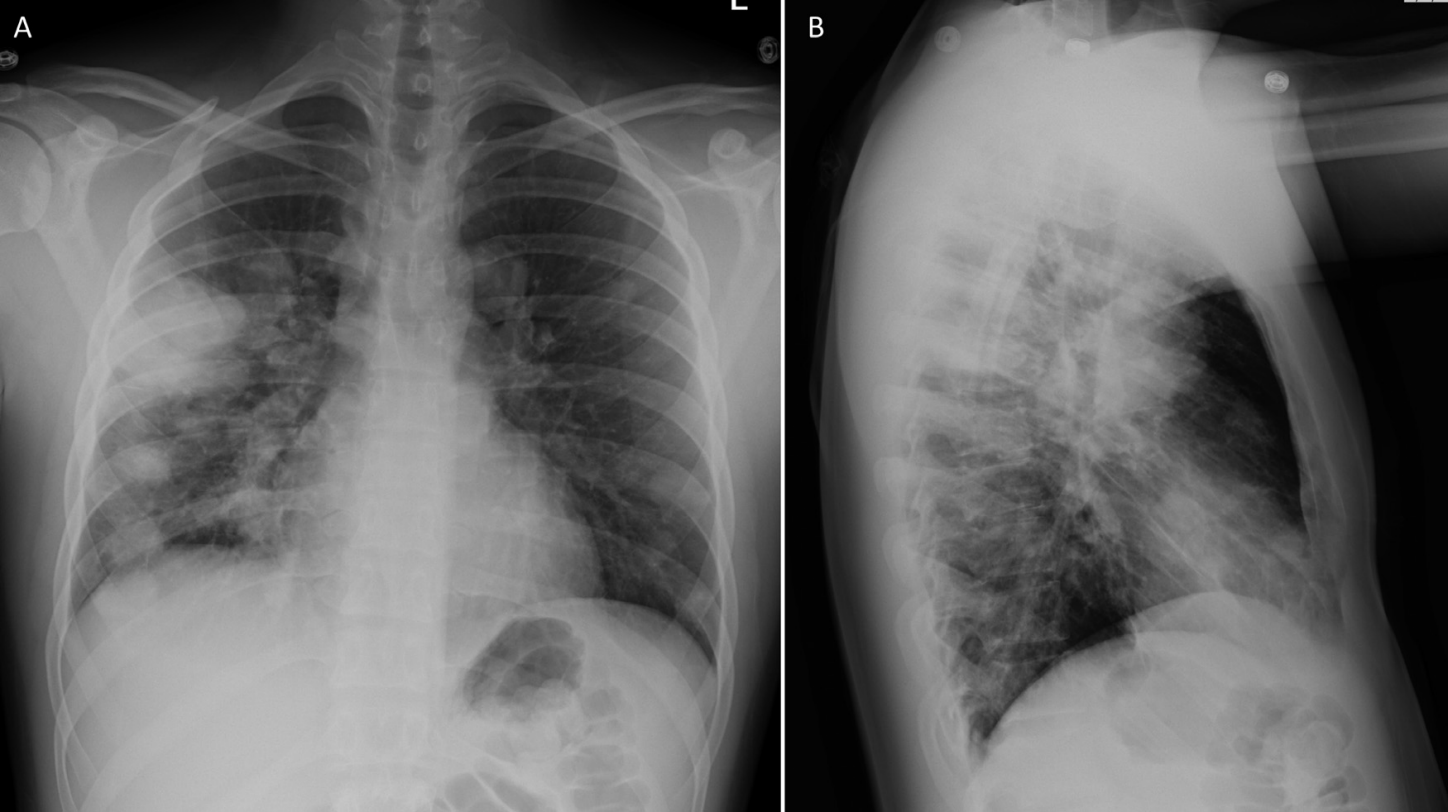
PA (A) and lateral (B) Chest radiograph demonstrates bilateral (right greater than left) multifocal nodular opacity, concerning for round pneumonia.

**Fig. 2:**
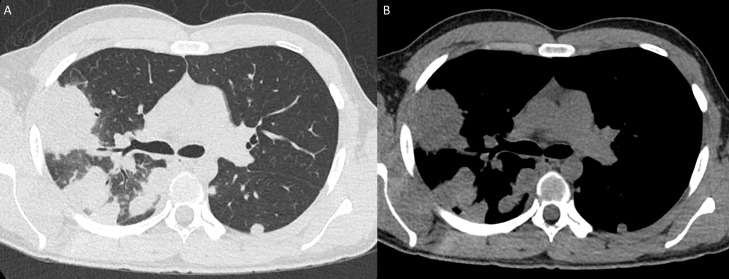
CT of the chest axial images, lung (A) and soft tissue (B) reconstruction algorithm, showed numerous (more than 30) bilateral pulmonary nodules/masses, ranging from 20-60 mm in diameter with associated peripheral halo. No pleural effusion or pneumothorax and patent central airway. Findings were favored to represent vasculitis.

**Fig. 3:**
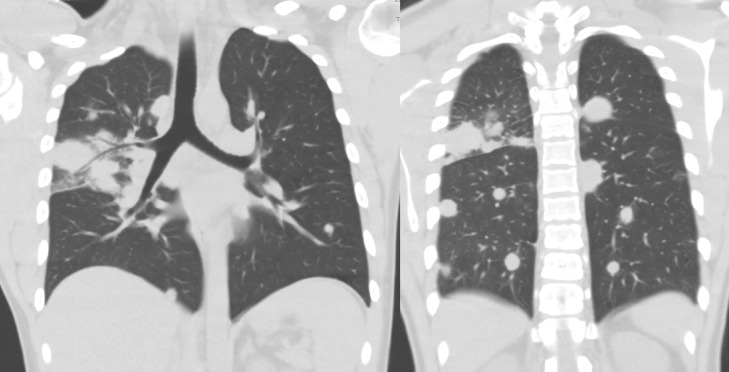
CT of the chest coronal images, showed numerous (more than 30) bilateral pulmonary nodules/masses, ranging from 20-60 mm in diameter with associated peripheral halo sign. No pleural effusion or pneumothorax and patent central airway. Findings were favored to represent vasculitis.

Chest radiograph was performed which showed bilateral (right greater than left) multifocal nodular opacity, concerning for round pneumonia (Fig. 1). Further evaluation with CT of the chest showed numerous (more than 30) bilateral pulmonary nodules/masses, ranging from 20-60 mm in diameter with associated peripheral halo . No pleural effusion or pneumothorax and patent central airways were noted (Figs. 2 and 3). Findings were favored to represent vasculitis.

**Fig. 4:**
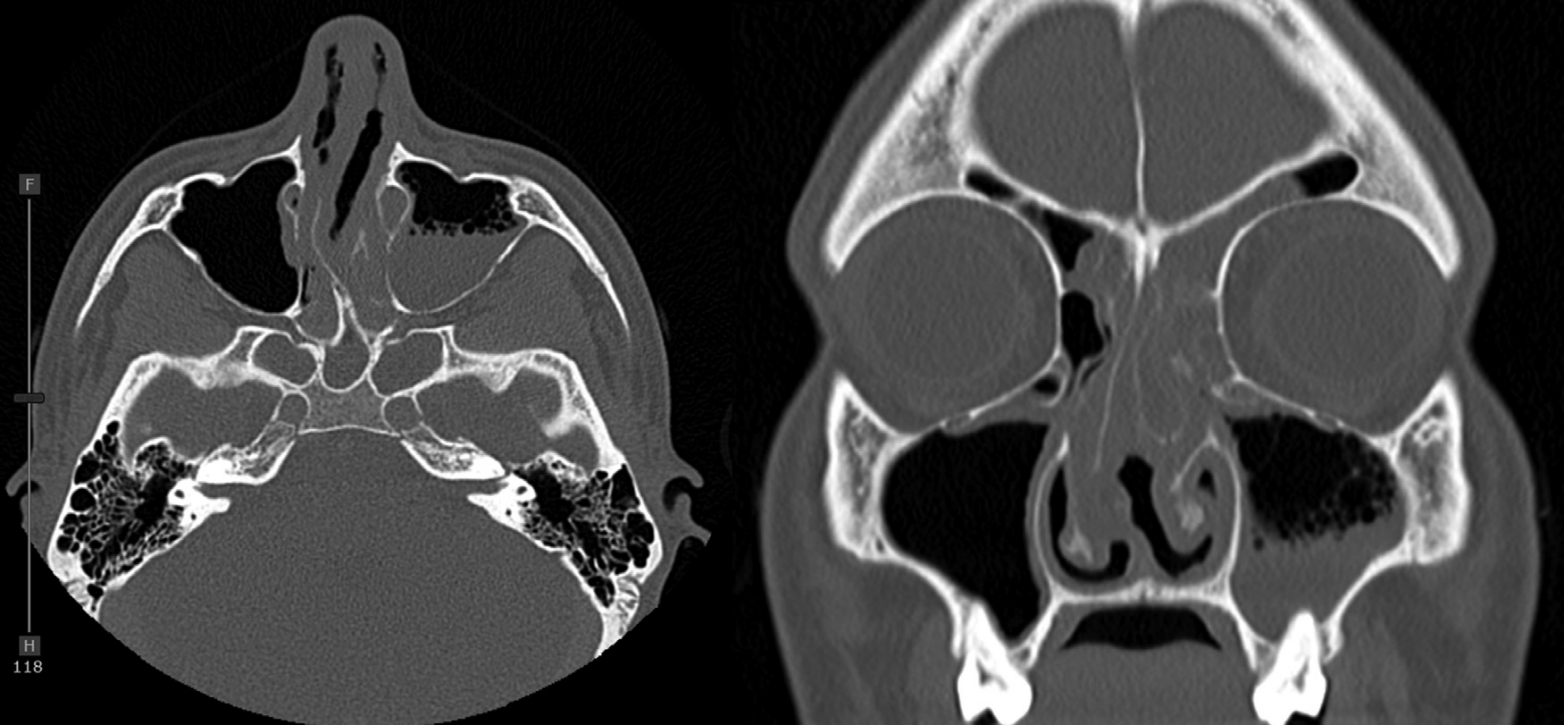
CT sinus protocol showed complete opacification of left frontal and left sphenoid sinus. Frothy secretions with air-fluid level in the left maxillary sinus. Moderate mucosal thickening of the right maxillary sinus and left greater than right anterior and posterior ethmoid sinuses.

**Fig. 5:**
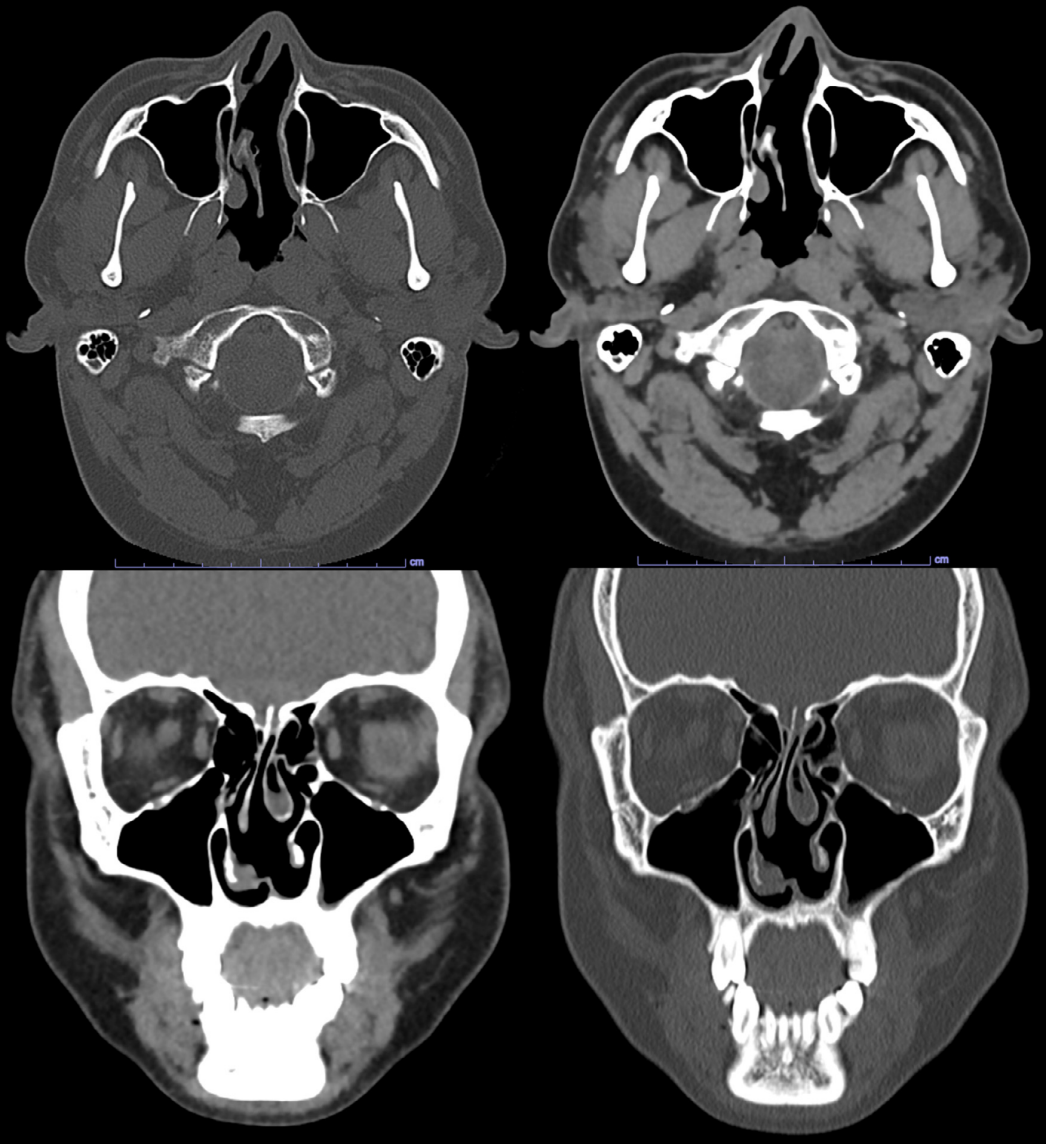
CT Sinus protocol demonstrates erosion of the anterior cartilaginous portion of the nasal septum

Unenhanced CT brain was performed for evaluation of headache which showed no acute intracranial abnormalities. Frothy secretions were seen in the left maxillary sinus, representing active sinus disease. Further evaluation with CT sinus protocol was performed which showed complete opacification of left frontal and left sphenoid sinus. Frothy secretions were again noted with air-fluid level in the left maxillary sinus. Moderate mucosal thickening of the right maxillary sinus and left greater than right anterior and posterior ethmoid sinuses (Fig. 4). Additionally, there was erosion of the anterior cartilaginous portion of the nasal septum (Fig. 5). Constellation of these findings were suggestive of c-ANCA vasculitis (granulomatosis polyangiitis, formerly Wegener's granulomatosis). Subsequently, serum C-ANCA was positive 1:128.

**Fig. 6:**
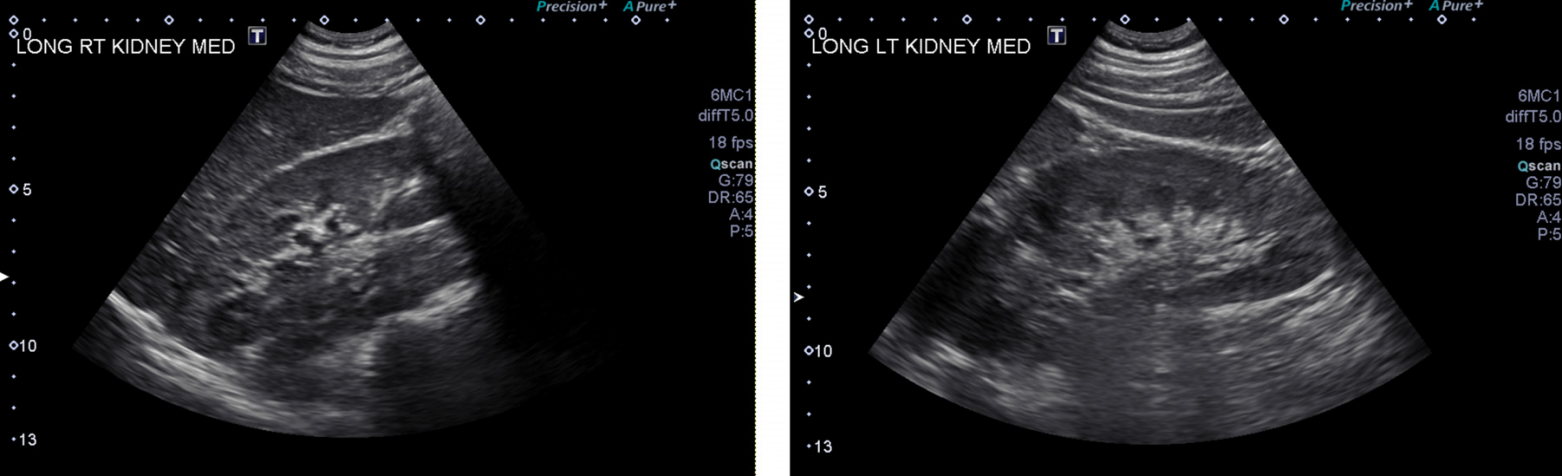
Normal sonogram of bilateral kidneys.

Further evaluation of the kidney with ultrasound revealed normal findings (Fig. 6).

**Fig. 7:**
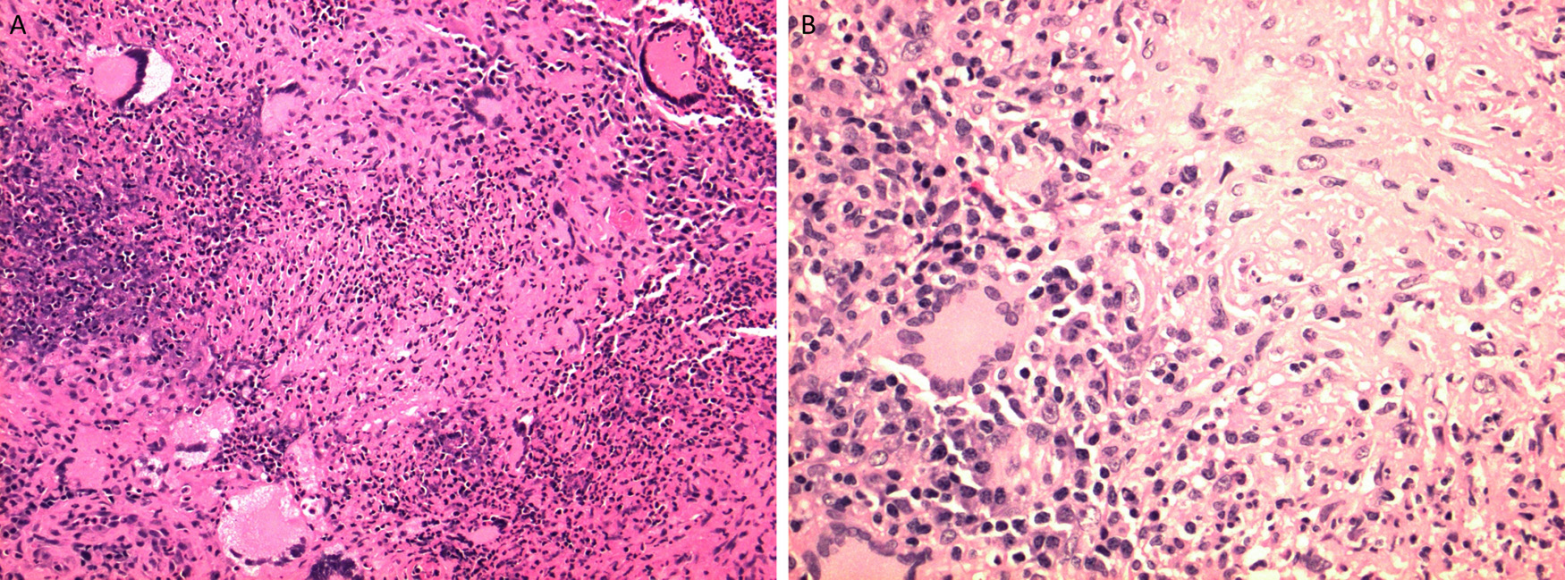
Hematoxylin and Eosin 200x (A) and 400x (B) sections of nasal septum showed numerous necrotizing granulomas and foreign-body type giant cells within a background of necroinflammatory debris Necrotizing granulomatous inflammation with foreign-body giant cells.

Evaluation of the H&E sections of the nasal biopsy showed numerous necrotizing granulomas and foreign-body type giant cells within a background of necroinflammatory debris. No acid–fast or fungal organisms are identified by immunohistochemical special stains which include Fite-Acid fast bacilli, Kinyoun's, Grocott-Gomori's methenamine silver stain and Warthin –Starry. Additional immunohistochemical stains to include CD3, CD20 and CD138 (T, B-cell and plasma cell markers respectively) showed no evidence of lymphoma. Carcinoma and metastatic malignancies were ruled out as well. Other potential differential diagnoses include an infectious etiology, although no organisms were identified on immunohistochemical, reaction to a foreign substance or material although no polarizable material was identified on polarized microscopy. The overall findings are supportive of a reactive/inflammatory process comprised of numerous necrotizing granulomas and foreign-body giant cells (Fig. 7).

## Discussion

Antineutrophil cytoplasmic antibody associated vasculitis includes a heterogeneous group of autoimmune conditions which are characterized by necrotizing vasculitis and positive ANCA titers. The titers are reactive to proteinase-3 (PR3-ANCA), C-ANCA or myeloperoxidase (MPO-ANCA) – pANCA [12]. These vasculitis can affect arterioles, capillaries or venules involving any organ system with varying degree of severity. The commonly involved organ system includes lungs and kidneys [2,4].

These vasculitis could be broadly categorized into granulomatosis with polyangiitis (positive to cANCA in 90% of cases), microscopic polyangiitis (positive to pANCA in 50%-90% of cases), idiopathic pauci-immune pulmonary capillaritis, idiopathic pauciimmune rapidly progressive glomerulonephritis and eosinophilic granulomatosis with polyangiitis (positive to cANCA in about 35%-75% of cases) [2,4,9,10,12].

Pulmonary vasculitis can be classified as small vessel or large vessel vasculitis [10]. Small vessel vasculitis includes ANCA associated vasculitis (granulomatosis with polyangiitis which includes ANCA associated pulmonary renal syndrome, microscopic polyangiitis and eosinophilic granulomatosis with polyangiitis), idiopathic pauci-immune pulmonary capillaritis, non-ANCA associated pulmonary renal syndrome which includes Goodpasture's syndrome, cryoglobulinemic vasculitis. The large vessel vasculitis includes Takayasu arteritis, giant cell arteritis, Behcet's disease and Hugh Stovin syndrome. Additionally, few granulomatous processes in the lung also have an angiocentric component which includes necrotizing sarcoid granulomatosis, bronchocentric granulomatosis, and lymphoid granulomatosis [6].

Pulmonary-renal syndromes refer to a group of condition which affects both lungs and kidneys. These conditions are typically characterized by diffuse alveolar hemorrhage and glomerulonephritis which includes pulmonary vasculitis, Goodpasture's syndrome, systemic autoimmune conditions including systemic lupus erythematosus, cryoglobulinemia and systemic sclerosis, antiphospholipid syndromes and iatrogenic drug-induced conditions [13].

Granulomatosis with polyangiitis, formerly known as Wegener granulomatosis is a multi-system necrotizing noncaseating granulomatous vasculitis affecting small-medium arteries, capillaries and veins. It has a male predilection and onset is typically around 50 years of age. Patient presents with cough, hemoptysis, chronic nasal obstruction/sinus symptoms, proteinuria/hematuria. The pathologic masses include immune mediated vascular injury. Histology demonstrates necrotizing granulomas with an associated vasculitis. 90% of the cases demonstrates positive cANCA (PR3), which also correlate with disease activity. The classic triad of organ involvement includes lung (involved in 95% of cases), upper respiratory tract/sinuses (involved in 75%-90% of cases), and kidneys (involved in 80% of cases) [2,4,9,10,12].

Based on organ system involvement granulomatosis with polyangiitis can be further classified as: Classical (which includes all 3 organs), limited (usually respiratory tract involvement only) and widespread which also involves additional organ system including skin (in 50% of cases), eyes (in 45% of cases) and peripheral nervous system (in 35% of cases) [2,4,9,10,12].

Management includes immunosuppressants (cyclophosphamide, methotrexate and steroids). Without treatment, granulomatosis with polyangiitis is rapidly progressive with 10% two-year survival. Appropriate medical therapy has dramatically increased long-term survival [4].

## Patient consent

Patient was lost to follow-up and informed consent was not possible to obtain. No patient identifiers are documented in this report.

## Disclosure

No relevant disclosures
